# Revealing the clinical relevance of *Staphylococcus borealis*

**DOI:** 10.1128/spectrum.01988-24

**Published:** 2025-03-12

**Authors:** Jorunn Pauline Cavanagh, Claus Klingenberg, Hermoine Jean Venter, Jan Egil Afset, Olaf Stromme, Paul Christoffer Lindemann, Therese Johansen, Kyriakos Zaragkoulias, Hege Vangstein Aamot, Ståle Tofteland, Pia Littauer

**Affiliations:** 1 Department of Clinical Medicine, Research Group for Child and Adolescent Health, UiT-The Arctic University of Norway, Tromsø, Norway; 2 Center for New Antibacterial Strategies, UiT-The Arctic University of Norway, Tromsø, Norway; 3 Department of Pediatrics, University Hospital of North Norway, Tromsø, Norway; 4 St. Olavs University Hospital, Trondheim, Norway; 5 Department of Clinical and Molecular Medicine, Faculty of Medicine and Health Sciences, Norwegian University of Science and Technology, Trondheim, Norway; 6 Department of Microbiology, Haukeland University Hospital, Bergen, Norway; 7 Department of Microbiology, The Nordland Hospital Trust, Bodø, Norway; 8 Department of Laboratory Medicine, Levanger Hospital, Nord-Trøndelag Hospital Trust, Levanger, Norway; 9 Department of Medical Microbiology, St. Olavs University Hospital, Trondheim, Norway; 10 Department of Microbiology and Infection Control, Akershus University Hospital, Lørenskog, Norway; 11 Department of Clinical Microbiology, Sorlandet Hospital Trust, Kristiansand, Norway; 12 Department of Microbiology and Infection Control, University Hospital of North Norway, Tromsø, Norway; University of Arizona College of Medicine Tucson, Tucson, Arizona, USA

**Keywords:** *Staphylococcus borealis*, urinary tract infections, skin and soft tissue infections, multidrug resistance, biofilm formation, clinical relevance

## Abstract

*Staphylococcus borealis*, previously misidentified as *Staphylococcus haemolyticus*, was first described as a new species in 2020. In this study, we aimed to describe the clinical relevance of *S. borealis* by combining clinical data, antibiotic susceptibility profiles, and biofilm formation in isolates obtained from hospitalized and non-hospitalized patients. We established a collection of 129 *S*. *borealis* isolates from 129 adult patients from seven Norwegian hospitals. We describe clinical data at the time of microbiological specimen collection. Antibiotic susceptibility and biofilm formation were tested using established methods. Of 129 isolates (37%), 48 were from patients admitted to a hospital, the remaining from outpatients. The median (IQR) age was 62 (51–78) years, and 85/129 (66%) of the isolates were from male patients. The majority (81/129, 63%) of the *S. borealis* isolates were isolated from urine cultures, followed by isolation from skin and soft tissue cultures (35/129, 27%), blood cultures (8/129, 6%), and two implant-associated infections (2/129, 2%). Resistance to ≥3 antibiotic classes was observed in 43/129 (33%) of the isolates. All isolates formed a biofilm under the conditions tested; 59/129 (46%) weak, 40/129 (31%) medium, and 29/129 (23%) were strong biofilm producers. *S. borealis* clinical samples were predominately obtained from elderly male patients, and the majority of samples were from patients with suspected urinary tract or skin and soft tissue infections. The level of multidrug resistance was comparable to other coagulase-negative staphylococcal species, but resistance toward methicillin and penicillin was lower than in clinical *S. haemolyticus* isolates.

**IMPORTANCE:**

This study contributes novel knowledge on the clinical relevance of *Staphylococcus borealis*; this is of importance when clinical microbiologists encounter *S. borealis* identified in patient samples. *S. borealis* was mainly identified in microbiological specimens from middle-aged to elderly patients, predominantly males. Hospitalized patients were also frequently immunocompromised and had other underlying conditions accompanying a suspected S. borealis infection.

## INTRODUCTION

Coagulase-negative staphylococci (CoNS) is a heterogeneous group of species within the genus *Staphylococcus*. They are a major cause of opportunistic infection in immunocompromised individuals and are associated with biofilm formation on indwelling medical devices. However, there are differences in both virulence and antimicrobial resistance (AMR) patterns within the CoNS group (1). *Staphylococcus borealis* was previously misidentified as *Staphylococcus haemolyticus*. However, based on 16S rRNA gene sequencing and matrix-assisted laser desorption ionization time-of-flight mass spectrometry (MALDI-TOF MS) analyses, marked differences between *S. haemolyticus* and *S. borealis* isolates were detected. Subsequently, following whole genome sequence analyses*, S. borealis* was described as a new member of the CoNS group in 2020. *S. borealis* is genotypically distinct from other CoNS, has a yellow-pigmented phenotype, and produces urease (2).

Some CoNS are associated with specific infection sites, like the urinary tract (*Staphylococcus saprophyticus*), or invasive infections, like endocarditis (*Staphylococcus lugdunensis*). Two recent publications have described that *S. borealis* may cause bovine mastitis (3, 4). Fecal carriage of *S. borealis* has also been reported in pigs (5) and in samples from healthy children (6). However, there is virtually no data on the clinical relevance of this new staphylococcal species in humans.

*S. haemolyticus* is ranked as the most antibiotic-resistant member of the CoNS group, and nosocomial infections are “difficult to treat” (7). Very little is known about AMR in *S. borealis,* but the first genomic analyses identified the presence of genes encoding resistance to multiple antimicrobial agents (2). Fecal isolates from humans (6) and pigs (5) were reported as multi-drug resistant (MDR), and the porcine isolates carried both SCCmec type V and the unusual macrolide resistance genes *ermT* and *erm 43* (5). The findings of *S. borealis* both in porcine and bovine samples suggest a zoonotic potential. However, the level of AMR in *S. borealis* isolates from humans is still unknown.

In 2021, the MALDI-TOF spectrum for *S. borealis* was incorporated in the updated Bruker database used by the MALDI biotyper. Clinical microbiologists will therefore now encounter *S. borealis* identified in patient samples. Without clinical background information, it is challenging to interpret the significance of finding *S. borealis*. In this study, we present data from a large (*n* = 129) collection of clinical *S. borealis* isolates identified from seven Norwegian hospitals. Our aim was to describe clinical data collected during infection episodes where *S. borealis* was detected and the results of detailed phenotypic studies of the antibiotic susceptibility and the biofilm-forming ability of *S. borealis*.

## MATERIALS AND METHODS

### Study design

This is a national multicenter study including seven clinical microbiology departments in Norwegian hospitals. We established a collection of 129 *S*. *borealis* isolates using both a retrospective and prospective approach. All positive blood culture isolates are routinely stored at the microbiological departments. In the retrospective part of the study, all clinical *S. haemolyticus* blood culture isolates that had been collected and stored between 2014 and 2021 in the participating hospitals were re-analyzed by MALDI-TOF MS (Bruker Daltonics).

We searched for isolates that, with the latest MALDI-TOF Bruker database update, 2021, would now be classified as *S. borealis* (score ≥2.0). In the prospective part, we searched for all *S. borealis* isolates identified between February 2021 and November 2022. All identified *S. borealis* isolates were transported to one central laboratory for further investigation. All *S. borealis* isolates, independent of them being considered as the main cause of infection or not, were stored for the purpose of this study.

### Clinical data

Clinical data were collected from the medical information accompanying the specimen sent to the clinical microbiology laboratories. These data included age, sex, source of specimen, hospital ward for admitted patients or whether they were treated as outpatient, clinically suspected type of infection, and if antibiotic therapy had been initiated (Table S1). We included only patients ≥ 18 years of age, and all patient data were anonymized.

### Antimicrobial susceptibility testing (AST)

AST was performed for this study by the disk diffusion method according to the EUCAST guidelines v.14.0 (8, 9). The following panel of antimicrobial agents was tested: fusidic acid (FD10), cefoxitin (FOX30), penicillin G (P1), erythromycin (E15), clindamycin (DA2), trimethoprim–sulfamethoxazole (SXT25), trimethoprim (TMP 5), nitrofurantoin (NFT 100), gentamicin (CN10), linezolid (LZD 10), ciprofloxacin (CIP 5), tetracycline (TE 30), and rifampicin (RD5) (Oxoid, Ireland). Vancomycin (0.016–256 μg/mL) was tested using the broth microdilution method (Liofilchem, Italy), according to the manufacturer’s guidelines. A cefoxitin inhibition zone <22 mm was interpreted as methicillin resistance, in line with EUCAST recommendations (10, 11). Multidrug resistance (MDR) was defined as resistance to ≥3 categories of antimicrobial agents. We interpreted AST data for *S. borealis* using breakpoints for other CoNS, as suggested by EUCAST (10).

### Biofilm formation

The biofilm-forming ability of the isolates was examined using the modified Christensen method (12). Briefly, bacterial overnight cultures were diluted 1:100 and grown in Tryptic Soy Broth (Bacto Tryptic Soy Broth, art. nr. 211825, BD, USA) with 1% glucose overnight in 96-well plates (Nunc Microwell, Thermo Scientific, Denmark) before staining with 0.1% crystal violet solution (Sigma Aldrich, Germany). After staining of the biofilm, the crystal violet was dissolved using 70% ethanol, and the optical density (OD) was read at 570 nm using ClariostarPlus (BMG Labtech, Germany). Three biological replicates using eight wells for each replicate were performed for all isolates. Biofilm formation of six urine catheter isolates and six skin and soft tissue isolates was tested using 60% artificial urine. The artificial urine had a pH of 6 ± 0.08 and was made according to Sarigul et al. (13). The strong biofilm producer *Staphylococcus epidermidis* RP62A and the poor biofilm producer *S. haemolyticus* 51-03 were used as positive and negative controls, respectively. After the removal of the highest and lowest values of eight replicates/parallels, the average OD values of three replicates were used.

### Phenotypic description

For phenotypic analyses of pigmentation and hemolysis, the isolates were streaked on horse blood agar plates (Thermofisher, Oxoid, UK) and on chocolate agar plates (Thermofisher Oxoid, UK) before incubation at 37°C for 12–18 h. Beta hemolysis was observed as a clear zone around the colonies.

### Statistical analysis

Data were analyzed using SPSS Statistics 29. Continuous variables were analyzed with a non-parametric test and proportions with a χ test. P-values less than 0.05 were considered significant.

## RESULTS

### Establishment of the *S. borealis* strain collection

Using the updated MALDI-TOF database for re-classification, we found 12 (3.9%) *S*. *borealis* isolates among 308 *S*. *haemolyticus* blood culture isolates that were re-tested in the retrospective part collected between 2014 and 2021. Seven of these 12 isolates were included in the study. The remaining 122 isolates in this study, from different body sites/sources, were collected in the prospective part of the study during 2021–2022.

### Clinical data

Table 1 shows baseline epidemiological and clinical data, including source of specimen and suspected type of infection, presented separately for hospitalized and non-hospitalized patients. The median (IQR) age of all patients was 62 (51-78) years, and 97/129 (75%) were ≥50 years of age. Overall, 85/129 (66%) isolates were from male patients. Most of the isolates, 81/129 (63%), were from patients receiving outpatient care. The majority of the *S. borealis* isolates were isolated from urine cultures 81/129 (62.8%), followed by isolates from skin and soft tissue specimens (35/129, 27.1%), blood cultures (8/129, 6.2%), and specimens from implant-associated infections 2 (1.6%). All isolates (*n* = 7) included from the prospective study were blood culture isolates.

**TABLE 1:** Source of specimen, infection type, and underlying conditions of patients with a positive identification of *S. borealis*

	All patients *N* = 129	Non-hospitalized *N* = 81	Hospitalized *N* = 48
Age, median (IQR), years	62 (51–78)	59 (40–75)	73 (58–81)
Male	85 (65.9%)	48 (59.3%)	37 (77%)
Specimen			
Urine	81 (62.8%)	57 (70.4%)	24 (50%)
Skin and soft tissue	35 (27.1%)	22 (27.2%)	13 (27.1%)
Blood culture	8 (6.2%)	0	8 (16.7%)
Other^*a*^	5 (3.9%)	2 (2.5%)	3 (6.3%)
Suggested type/focus infection			
Urinary tract infections	50 (38.9%)	36 (44.4%)	14 (29.1%)
Skin and soft tissue infections	15 (11.6%)	13 (19.8%)	2 (4.2%)
Sepsis	7 (5.4%)	0	7 (14.5%)
Implant-associated infection^*b*^	2 (1.6%)	0	2 (4.2%)
Airway infections	2 (1.6%)	0	2 (4.2%)
Other^*c*^	11 (8.5%)	7 (8.6%)	4 (8.3%)
No verified infection	43 (33.3%)	24 (29.6%)	19 (39.6%)
Immunocompromised			
Not reported	29 (22.5%)	21 (25.9%)	8 (16.7%)
No	89 (68.9%)	59 (72.8%)	30 (62.5%)
Yes	11 (8.5%)	1 (1.2%)	10 (20.8%)

^*a*^ Ear secretion, expectorate, abortion.

^*b*^ Out of in total six reported patients with implants, only two had an implant-associated infection.

^*c*^ External otitis, abdominal pain.

In total, 9/35 (25.7%) of samples from suspected skin and soft tissue infections (SSTIs) were from genital wounds. Suspected urinary tract infections (UTIs) and SSTIs were more common in non-hospitalized patients, in contrast to sepsis and implant-associated infections, which only occurred in hospitalized patients. Three of the patients with a UTI were reported to have a urinary catheter, and in total, six patients had some type of indwelling catheter. Of the hospitalized patients, 10/48 (20.8%) were reported to be immunocompromised, and two of the patients reported to be immunocompromised also had growth of *S. borealis* in the blood culture.

### Antimicrobial susceptibility profile

The AST profiles are presented in Table 2 and Table S2. All isolates were susceptible to vancomycin and linezolid. Rates of resistance to nitrofurantoin, tetracycline, and rifampicin were below 10% in both hospitalized and non-hospitalized patients. The highest rates of resistance were seen towards penicillin, erythromycin, and ciprofloxacin. Overall, 27/129 (20.9%) isolates were identified as methicillin-resistant. Inducible resistance to clindamycin was observed in 7/129 (5.4%) isolates. In total, 43/129 (33.3%) of the isolates were classified as MDR, with a higher proportion of MDR isolates from hospitalized (50%) versus non-hospitalized (23.5%) patients. MDR was not associated with any particular type of infection.

**TABLE 2:** Antimicrobial susceptibility, tested by the disk diffusion method, of 129 *S. borealis* isolated from non-hospitalized and hospitalized patients. The table is showing the number of resistant isolates

Antimicrobial agent	Non-hospitalized *N* = 81	Hospitalized *N* = 48	*P-*value
Fusidic acid	21 (25.9%)	12 (25%)	0.90
Cefoxitin ^*a*^	10 (12.3%)	17 (35.4%)	**0.0018^*d*^**
Penicillin	20 (24.6%)	25 (52.1%)	**0.0016**
Erythromycin	35 (43.2%)	16 (33.3%)	0.26
Clindamycin ^*b*^	26 (32.1%)	17 (35.4%)	0.69
Trimethoprim–sulfamethoxazole	0	2 (4.2%)	0.28
Gentamicin	4 (4.9%)	6 (12.5%)	0.12
Linezolid	0	0	
Ciprofloxacin	14 (7.3%)	23 (47.9%)	**0.0002**
Tetracycline	6 (7.4%)	1 (2.1%)	0.19
Rifampicin	3 (3.7%)	3 (6.3%)	0.50
Nitrofurantoin	1 (1.3%)	1 (2.1%)	0.70
Trimethoprim	7 (8.6%)	10 (20.8%)	**0.047**
Vancomycin	0	0	
MDR^*c*^	19 (23.5%)	24 (50%)	0.17

^*a*^ Cefoxitin used as indicator of methicillin resistance.

^*b*^ Inducible resistance to clindamycin was observed in 7/129 (5.4%) of the isolates.

^*c*^ MDR: Multidrug resistance, defined as resistance to ≥3 categories of antimicrobial agents.

^*d*^ Bold represents statistically significant values.

### Biofilm formation

Biofilm formation in TSB containing 1% glucose was observed for all isolates, apart from one isolate that did not grow in broth. The strong biofilm producer *S. epidermidis* RP62A formed biofilm yielding an average OD value of 1.7, while the poor biofilm producer *S. haemolyticus* 51-03 formed biofilm with an average OD value of 0.8. Based on the OD values of the reference strains, the *S. borealis* strains were classified as weak biofilm producers (OD 0.1–0.7), medium biofilm producers (OD 0.8–1.7), and strong biofilm producers (OD >1.7). Of the 128 tested *S. borealis* isolates, 59 (46.1%) were weak biofilm producers, 40 (31.3%) were medium biofilm producers, and 29 (22.3%) were strong biofilm producers under the experimental conditions used (Fig. S1). When tested under conditions that included artificial urine, biofilm formation was less pronounced for all 12 isolates, including the positive control, compared with results from the modified Christensen method. Only one urine culture isolate remained a strong biofilm producer when tested in artificial urine.

### Phenotypic description

**Fig 1:**
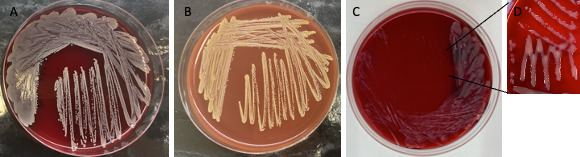
Phenotypic variation of *S. borealis* colonies. (A) *S. borealis* grown on blood agar plates. (B) *S. borealis* colonies on chocolate agar plates. (C and D) The mucoid phenotype observed.

Phenotypically, *S. borealis* isolates displayed a grayish white to yellow pigmentation on blood agar plates, with a more pronounced yellow pigmentation on chocolate agar media. Of the 129 isolates, 98 (76%) displayed yellow colonies on chocolate agar, while 31 (24%) displayed white colonies on chocolate agar (Fig. 1). There was no correlation between pigmentation and isolation site. On blood agar plates, a clear β-hemolytic zone was seen in all isolates after incubation for 24 h at 37°C. One clinical isolate had a mucoid colony morphology (Fig. 1). This isolate was from a post-operative bacteremia episode following resection of a bladder tumor. The mucoid colony was re-analyzed by MALDI-TOF and confirmed as *S. borealis.*

## DISCUSSION

In this study, *S. borealis* was mainly identified in microbiological specimens from middle-aged to elderly patients, predominantly males. Hospitalized patients were also frequently immunocompromised and/or had different underlying conditions, such as cancer, kidney transplant, or wounds accompanying a suspected *S. borealis* infection. These findings are well in line with the “high-risk population” of patients also seen in other opportunistic CoNS infections (1).

The main clinical finding is that *S. borealis* was most frequently identified in urine cultures and possible UTIs. However, the clinicians interpreting the microbiology results were often not yet aware of the potential clinical relevance of identifying *S. borealis*. As such, they may have considered the results as contamination and not an infection. Moreover, information on antibiotic treatment was not given for all possible UTI cases, making a further interpretation of the severity of infection difficult. The positive urine cultures were observed in individuals with serious underlying conditions, such as kidney transplant and kidney failure, but also in individuals with no underlying condition and in pregnant women. *S. saprophyticus* is known to cause uncomplicated UTIs, predominantly in young and middle*-*aged female patients (14, 15), but other CoNS have usually not been perceived as uropathogens (16). However, in a study from Tanzania, *S. haemolyticus* and *S. epidermidis* were also frequently identified in UTIs (17). *S. saprophyticus* has few virulence factors but produces urease, adhesins to uroepithelial cells, and transport systems involved in osmotolerance (15, 18). *S. borealis* also produces urease, a virulence factor associated with UTIs not only in S*. saprophyticus,* but also in other uropathogenic species, such as *Proteus mirabilis* (19) and *Klebsiella pneumoniae* (20, 21).

In our study, ~30% *S. borealis* isolates were from skin and soft tissue samples. CoNS colonize skin and mucosal surfaces (22). However, they rarely cause SSTIs, except in elderly or immunosuppressed patients, who may present with abscesses and sometimes vulval infections (23). In a recent study from Nigeria, *Staphylococcus cohnii*, *Staphylococcus condimentii*, *Staphylococcus sciuri*, and *Staphylococcus saprophyticus* were identified in 91/265 (34%) of skin and soft tissue samples (24). The high number of *S. borealis* isolates identified from skin and soft tissue samples in our study, and in particular associated with genital wounds, is interesting. However, we lacked detailed data on other bacteria detected during the same infection episode, and we did not have pathology reports that could elucidate on invasiveness. We could therefore not clearly resolve whether *S. borealis* was a contaminant, part of a mixed infection, or the main cause of the SSTI.

MDR was observed in 1/3 of all clinical *S. borealis* isolates. Even higher MDR rates were found in samples from hospitalized patients, in line with data on *S. haemolyticus* (2, 25) and other CoNS species (26). The high AMR rates against erythromycin and fusidic acid were comparable to results when mapping AMR in commensal CoNS isolates in Germany (27). However, when compared with a study mapping MDR (5.2%) in commensal CoNS isolates in Norway, the MDR rates (23.5%) were higher in *S. borealis* isolated from non-hospitalized patients (22). Methicillin resistance was only observed in 21% of *S. borealis* isolates from hospitalized patients compared with 70%–100% in clinical *S. haemolyticus* isolates (28, 29). The presence of MDR in *S. borealis* potentially adds on to the role of CoNS as resistance gene reservoirs for other CoNS and the more pathogenic *S. aureus* (30–32). The AST results should be interpreted with caution, as there is limited data on AST and clinical effects of antimicrobial agents for infections caused by* S. borealis.* Depending on the frequency of identification, clinical significance, and perceived clinical need, breakpoints for *S. borealis* may be established by EUCAST in the future (33).

All *S. borealis* isolates formed biofilm *in vitro* using a standard biofilm assay, and around 20% formed more biofilm than the strong biofilm producer *S. epidermidis* RP62a. Biofilm formation, primarily on medical implants, is one of the hallmarks of “difficult to treat” CoNS infections (1, 34), but we identified only two patients with a suspected *S. borealis*-associated medical implant infection and three patients with urinary tract catheters. However, biofilm formation is also a well-known virulence factor for many uropathogens and considered an important mechanism behind high rates of recurrent UTIs in some patients (35). Further testing of *S. borealis* biofilm formation using artificial urine did not reveal an improved biofilm forming ability; however, conditions could have been further optimized to also include catheter material.

This study has several strengths. It is the largest collection to date of *S. borealis* isolates presented with clinical data. The broad AST and biofilm testing were performed with established methods and give updated information on this newly described species. There are also several limitations. We used clinical data from the medical information accompanying the specimen sent to the clinical microbiology laboratories. However, these data often lacked the granularity required to clearly establish a causal relationship between the culture result and a clinical infection. Moreover, the study was designed as an observational study, not designed to determine causality. Additionally, since *S. borealis* was not a known pathogen during the study, the potential clinical relevance of identifying *S. borealis* was unclear to clinicians. This hampers the interpretation of some of our clinical results. However, our study indicates that *S. borealis* is a novel CoNS uropathogen. The clinical relevance of *S. borealis* in other types of infections needs to be further explored in large clinical studies with more detailed clinical descriptions. However, it seems to have a role in SSTIs and potentially invasive bacteremia in immunocompromised patients (36, 37). Ethical approval for our study included an opt-out approach, and we decided only to contact adults who were able to respond in person. Thus, lack of information on the relevance of *S. borealis* in children is also a limitation.

### Conclusion

In this first study describing the potential clinical relevance of *S. borealis,* we have described that this new staphylococcal species was mainly found in clinical samples from elderly males in specimens obtained due to suspected UTIs and SSTIs. The level of MDR in *S. borealis* is comparable to other CoNS, but resistance toward methicillin and penicillin is notably lower than what is observed in clinical *S. haemolyticus* isolates.

## SUPPLEMENTAL MATERIAL

Fig. S1 spectrum.01988-24-s0001.tiff Biofilm formation of *S. borealis* isolates.

Supplemental material spectrum.01988-24-s0002.docx Supplemental figure and table legends.

Table S1 spectrum.01988-24-s0003.docx Clinical questions asked.

Table S2 spectrum.01988-24-s0004.xlsx Results from antimicrobial susceptibility testing by disc diffusion.

ASM does not own the copyrights to Supplemental Material that may be linked to, or accessed through, an article. The authors have granted ASM a non-exclusive, world-wide license to publish the Supplemental Material files. Please contact the corresponding author directly for reuse.
