# Supplementary figures and images for: Revealing the clinical relevance of Staphylococcus borealis
Source: Microbiol Spectr. 2025 Mar 12;13(4):e01988-24. doi: 10.1128/spectrum.01988-24 (PMC11960051; doi:10.1128/spectrum.01988-24)

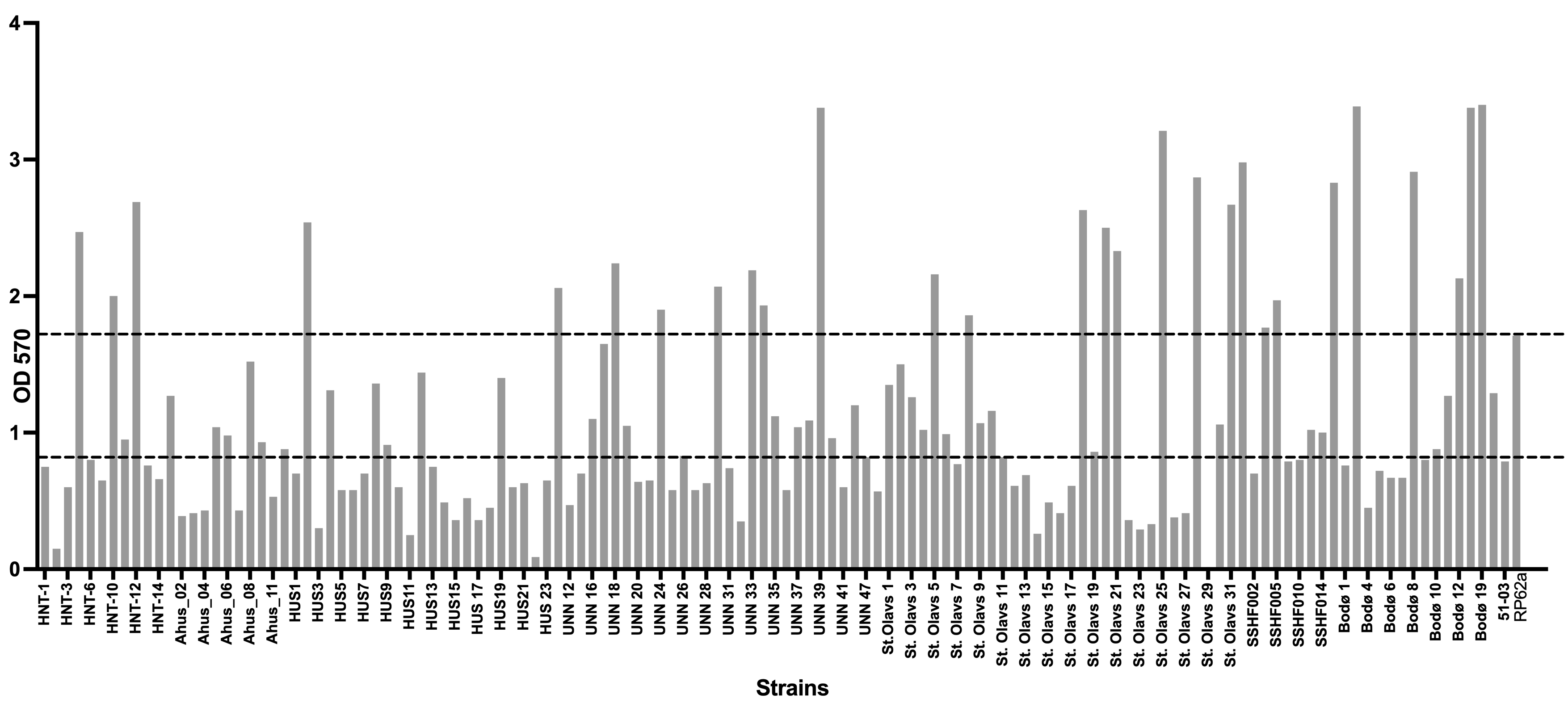

Supplement: Fig. S1 — Biofilm formation of S. borealis isolates. [file spectrum.01988-24-s0001.tiff]
