# Supplementary material for: Revealing the clinical relevance of Staphylococcus borealis
Source: Microbiol Spectr. 2025 Mar 12;13(4):e01988-24. doi: 10.1128/spectrum.01988-24 (PMC11960051; doi:10.1128/spectrum.01988-24)
Supplement: Supplemental material — Supplemental figure and table legends. [file spectrum.01988-24-s0002.docx]

**Supplementary Figure 1:** Biofilm forming ability of 128 *S. borealis* isolates. The horizontal lines indicate the cut off for weak, medium and strong biofilm formers based on the negative control, *S. haemolyticus* 51-03, and the positive control *S. epidermidis* RP62a.

**Supplementary Table 1:** Clinical data that clinicians were asked to extract from available patient data related to a positive *S. borealis* identification.

**Supplementary Table 2:** Zone diameter values for antimicrobial susceptibility testing by disc diffusion test.
