# Supplementary material for: Revealing the clinical relevance of Staphylococcus borealis
Source: Microbiol Spectr. 2025 Mar 12;13(4):e01988-24. doi: 10.1128/spectrum.01988-24 (PMC11960051; doi:10.1128/spectrum.01988-24)
Supplement: Table S1 — Clinical questions asked. [file spectrum.01988-24-s0003.docx]

|  |  |
| --- | --- |
| 1 | Which ward at your hospital was the patient submitted to when experiencing a *S. borealis*infection? ​ |
| 2 | What type of infection was *S. borealis*associated with? |
| 3 | In which test material was *S. borealis*identified? ​ |
| 4 | What was the patient's primary diagnosis? |
| 5 | Was the patient immunocompromised? Did they use immunosuppressants? |
| 6 | Was this a catheter related infection? |
| 7 | Had the patient received antimicrobial agents prior to the*S. borealis*infection? |
| 8 | Was the patient treated with antimicrobial agents due to the *S. borealis*infection? ​ |
| 9 | For how long was the patient hospitalized in relation to the infection where *S. borealis*was identified? ​ |
| 10 | Was *S. borealis* considered the main cause of the disease episode? |
